# Leader gene identification for digestive system cancers based on human subcellular location and cancer-related characteristics in protein–protein interaction networks

**DOI:** 10.3389/fgene.2022.919210

**Published:** 2022-09-26

**Authors:** Hongwei Chen, Zherou Rong, Luanfeng Ge, Hongzheng Yu, Chao Li, Manyi Xu, Zihan Zhang, Junjie Lv, Yuehan He, Wan Li, Lina Chen

**Affiliations:** College of Bioinformatics Science and Technology, Harbin Medical University, Harbin, China

**Keywords:** cancer leader gene, digestive system cancer, protein–protein interaction network, subcellular location, cancer-related characteristics

## Abstract

Stomach, liver, and colon cancers are the most common digestive system cancers leading to mortality. Cancer leader genes were identified in the current study as the genes that contribute to tumor initiation and could shed light on the molecular mechanisms in tumorigenesis. An integrated procedure was proposed to identify cancer leader genes based on subcellular location information and cancer-related characteristics considering the effects of nodes on their neighbors in human protein–protein interaction networks. A total of 69, 43, and 64 leader genes were identified for stomach, liver, and colon cancers, respectively. Furthermore, literature reviews and experimental data including protein expression levels and independent datasets from other databases all verified their association with corresponding cancer types. These final leader genes were expected to be used as diagnostic biomarkers and targets for new treatment strategies. The procedure for identifying cancer leader genes could be expanded to open up a window into the mechanisms, early diagnosis, and treatment of other cancer types.

## Introduction

Digestive system cancers represent an important cause of mortality worldwide. Of these cancer types, colon, liver, and stomach cancers are among the top five common leading causes of cancer deaths in the world according to the International Agency for Research on Cancer (https://www.iarc.who.int/wp-content/uploads/2020/12/pr292_E.pdf).

To better understand the molecular mechanisms of cancers and develop the paradigm and biomarkers of targeted anticancer therapies, it is critical to accurately identify important genes that contribute to cancer initiation and development (Tamborero et al., 2018; Colaprico et al., 2020). Several computational methods have been developed for finding these kinds of genes. Some of these methods rely on mutations in genomic data or expression data of cancer cell lines. For example, Bailey et al. (2018) performed a pan-cancer and pan software analysis on point mutations and small indels in cancer genomic datasets, identifying 299 cancer driver genes. Ding et al. (2021) gave a computational investigation on expression data of cancer cell lines and discovered a new set of potential biomarkers of different cancers. Other methods identify genes on the basis of their proximity to cancer genes from various evidence, such as biomolecular networks. Liu et al. (2022) employed a model-free computational method to not only identify the critical transition states of ten cancers but also provide new biomarkers from a network perspective. Nie et al. (2020) constructed a protein–protein interaction (PPI) network for differentially expressed genes (DEGs) between stomach cancer and normal tissues and identified ten hub genes highly related to stomach cancer. Furthermore, the Cox regression model showed high expression of five genes that were significantly associated with late-stage stomach cancer. Hu et al. (2020) discovered three most significant genes involved in liver cancer progression by examining signaling pathway networks. In a study by Zhao et al. (2019), a PPI network was constructed for DEGs of colon cancer patients, and the hub genes were identified as potential key genes. Based on related research studies on PPI networks and graph theory, it was assumed that a set of genes could affect the entire network by affecting the expression of their neighbor genes through interactions (Wuchty, 2014). Of these genes, those that were directly involved in the occurrence of cancer were defined as cancer leader genes in this article.

The subcellular location of gene products is fundamental for understanding their functions in biological processes (Su et al., 2020). Li et al. (2017) reported that dysregulation of USP9X, an integral component of centrosome and a requisite for centrosome biogenesis, contributed to centrosome amplification, chromosome instability, and breast cancer. TMEM106B is a transmembrane protein located on cellular lysosomes. Kundu et al. (2018) demonstrated that TMEM106B-induced lysosomes released active lysosomal cathepsins necessary for cancer cell invasion and metastasis. Thus, in this article, on the basis of the disease network obtained from PPI data, subcellular location-specific networks were constructed with the subcellular location information. Based on the definition of a leader gene set, a graph theory-based algorithm was proposed and used to recognize the candidate leader genes in each network. Then, cancer-related characteristics, including hallmarks, gene functions, and classification performance, were considered to identify cancer leader genes. The results of the disease network and the subcellular location-specific ones were compared. The union of the leader genes from all networks was the final result of this article. This integrated procedure was conducted on three digestive system cancers, namely, stomach cancer, liver cancer, and colon cancer, to identify their leader genes.

## Materials and methods

**FIGURE 1:**
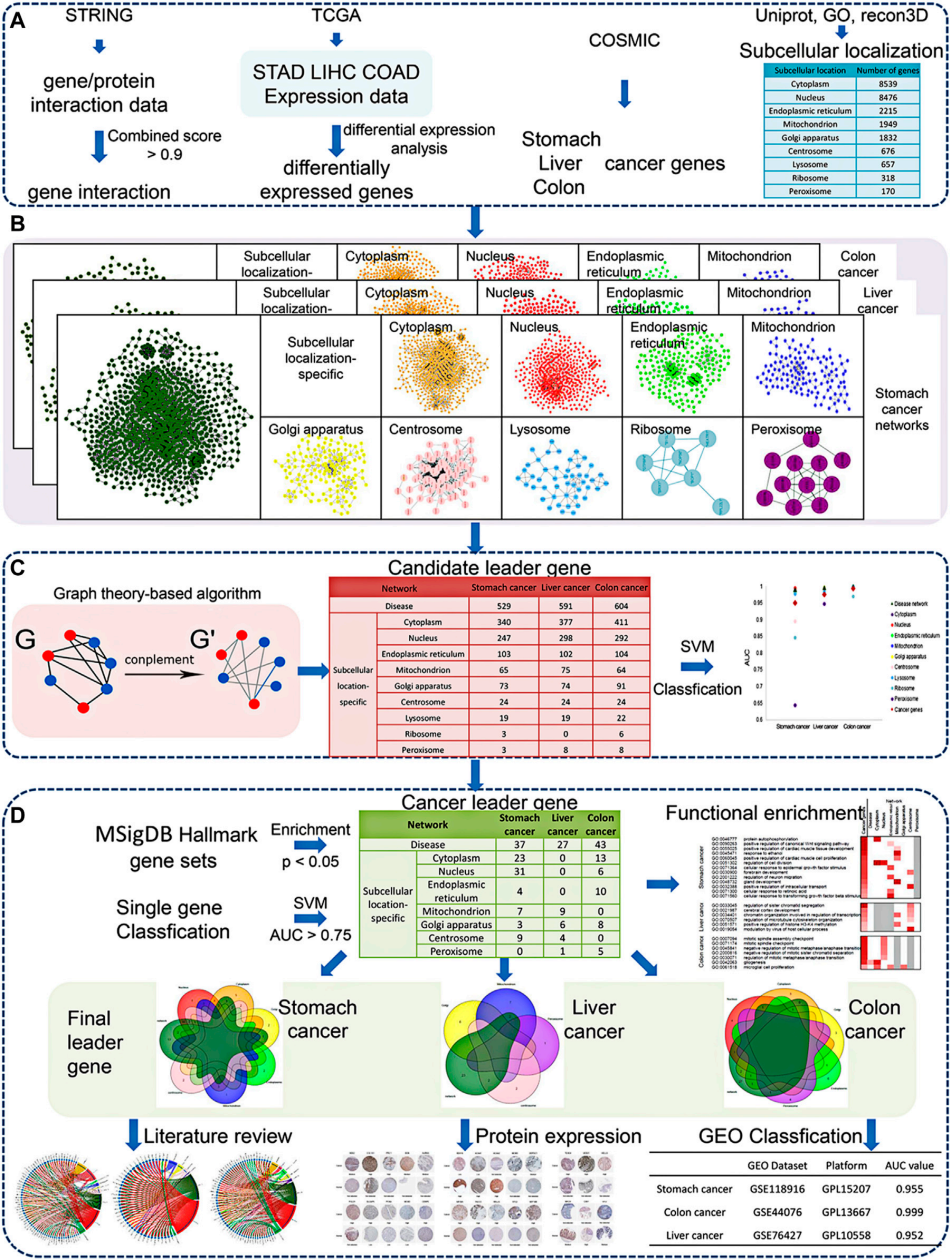
Workflow of our methodology. **(A)** Data sources. **(B)** Disease network construction. **(C)** Candidate leader gene recognition. **(D)** Leader gene identification and validation.

The procedure of our article is shown in Figure 1, and the details are described in the following sections

### Data

In this study, publicly available expression data for stomach adenocarcinoma (STAD), liver hepatocellular carcinoma (LIHC), and colon adenocarcinoma (COAD) cohorts were obtained from The Cancer Genome Atlas (TCGA, https://portal.gdc.cancer.gov/) for analysis. The stomach cancer cohort contained 407 samples (375 cancer and 32 normal samples), liver cancer contained 424 samples (371 cancer and 53 normal samples), and colon cancer contained 521 samples (478 cancer and 43 normal samples). Differential expression analysis was performed using the DESeq2 R package (adjusted *p*-value <0.05 and |log_2_FC| > 1). Also, 5797, 5240, and 6645 DEGs were obtained for stomach, liver, and colon cancers, respectively.

PPI data were retrieved from the STRING database (https://string-db.org/) (Szklarczyk et al., 2019). A total of 392,028 interactions between 9,588 gene products with a combined score >0.9 were obtained. In the following sections, they were treated as gene interactions.

Gene subcellular location information was obtained from UniProt, Gene Ontology (GO, http://geneontology.org/) (Gene Ontology, 2021), and Recon3D (http://vmh.life) (Table 1) (Brunk et al., 2018). Genes located in nine main subcellular locations were used in this study.

**TABLE 1:** Number of genes in different subcellular locations.

Subcellular location	Number of genes
Cytoplasm	8539
Nucleus	8476
Endoplasmic reticulum	2215
Mitochondrion	1949
Golgi apparatus	1832
Centrosome	676
Lysosome	657
Ribosome	318
Peroxisome	170

Cancer genes were downloaded from the Cancer Gene Census (http://cancer.sanger.ac.uk/census) (Kundu et al., 2018). For stomach, liver, and colon cancers, 24, 13, and 15 genes were retrieved, respectively.

### Disease and subcellular location-specific network construction

By using the gene interactions as edges and DEGs and cancer genes as nodes, a disease network for stomach cancer, liver cancer, and colon cancer was constructed. Considering gene subcellular location information, nine subcellular location-specific networks for each of these three cancer types were filtered out from the disease network by extracting interactions between genes of specific subcellular locations, respectively.

### Candidate leader gene recognition

**FIGURE 2:**
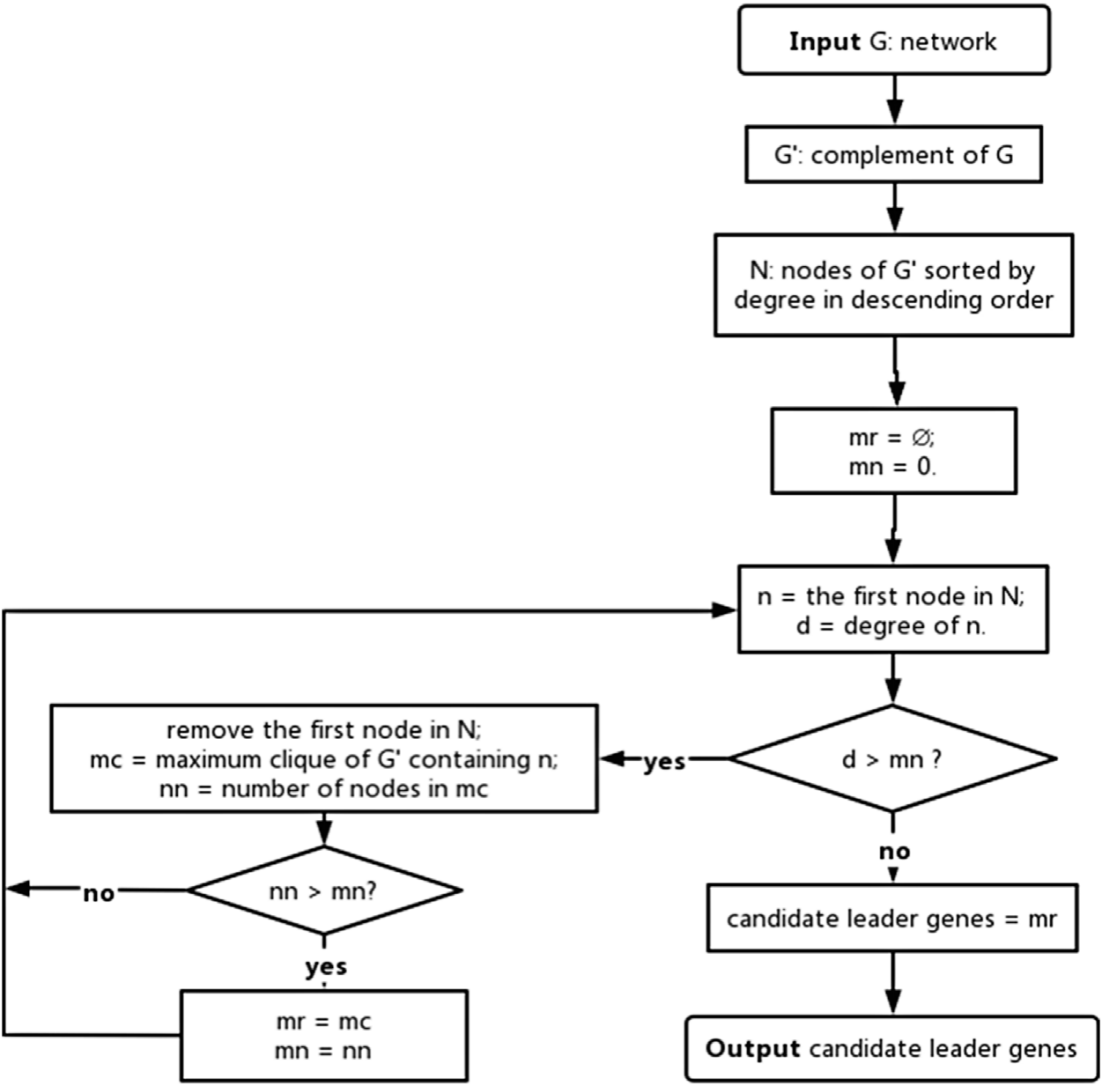
Graph theory-based algorithm for candidate leader gene recognition.

Cancer leader genes affect the entire network by influencing their neighbors in the network. The maximum independent dominant set in the graph theory has similar properties as leader genes in networks. Therefore, based on two theorems in the graph theory, the maximal independent set of a graph must be its minimal dominating set that affects the entire network, and the largest clique of a graph must be the largest independent set of its complement, which is a graph that has the same points as an original graph, and these points are connected by edges if and only if they are not connected by edges in the original graph. A graph theory-based algorithm was proposed to recognize genes that affect all nodes in the networks (Figure 2), which were referred to as candidate leader genes.

### Leader gene identification and validation

Leader genes were identified from candidate genes based on cancer-related characteristics. On the one hand, cancer-related genes were supposed to affect primarily a handful of essential cellular functions, termed cancer hallmarks (Hanahan and Weinberg, 2000). Therefore, enrichment analysis was conducted on each candidate leader gene set recognized from the networks. Enrichment categories were cancer hallmarks (50 hallmark gene sets from the Molecular Signatures Database (MSigDB)) (Liberzon et al., 2015) and biological processes enriched by cancer genes. Gene functional annotation was carried out with the clusterProfiler R package.

On the other hand, cancer leader genes contributing to tumor initiation should be able to classify samples of cancer/normal status. Here, a support vector machine (SVM) classifier was built to distinguish between cancer and normal samples using the expression of leader genes for cancer/normal samples from TCGA data as features. A leave-one-out cross-validation (LOOCV) was used to evaluate the overall performance of the classifier. Then, a receiver operating characteristic (ROC) curve was plotted, and the value of the area under the curve (AUC) was calculated to evaluate the classification performance.

Final leader genes were identified as those enriched in cancer hallmarks and cancer-related functions (BH-adjusted *p* < 0.05) and had good classification performance (AUC >0.75). Final leader genes were evaluated using the literature review, which was searched in the PubMed database (https://pubmed.ncbi.nlm.nih.gov/). To demonstrate the clinical significance of final leader genes, expression differences for gene products and classification performance of final leader genes for other datasets were analyzed. Expression levels for proteins encoded by final leader genes were obtained from the Human Protein Atlas (HPA, https://www.proteinatlas.org/) database (Wang et al., 2020a), which is an online server containing the human transcriptomic and proteomic data in cells, tissues, and organs from human normal or pathological tissues *via* immunohistochemistry (IHC) (Uhlen et al., 2017). The pathology section of HPA shows the protein levels of cancer patients. Expression levels for proteins encoded by final leader genes in normal and cancer tissues could be obtained in HPA by using gene names as the search terms. The expressional levels of the gene products were denoted as high, medium, low, or not detected as the combination of staining intensity and fractions of stained cells based on immunohistochemical data. Expression values of final leader genes were also used to classify samples from three independent datasets from the Gene Expression Omnibus (GEO, https://www.ncbi.nlm.nih.gov/geo/) database (Barrett et al., 2013).

## Results

### Subcellular location-specific networks

**FIGURE 3:**
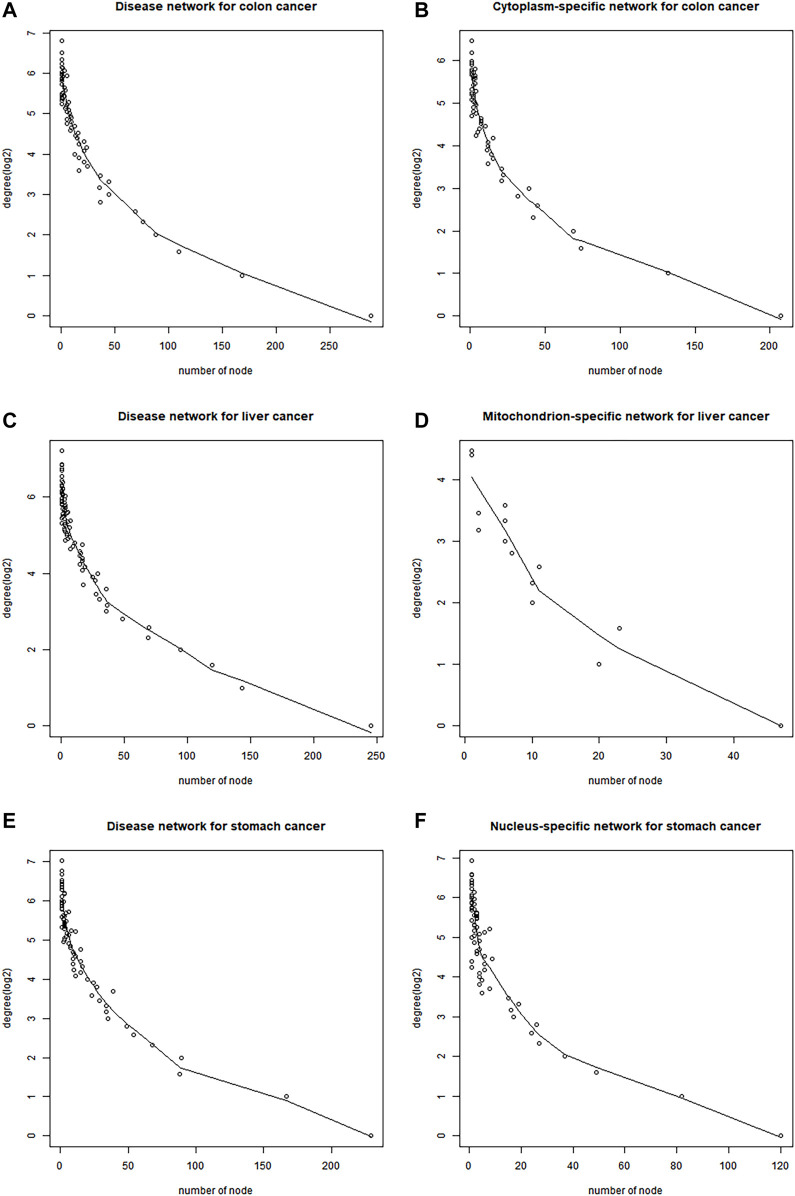
Degree distribution for part of disease networks and subcellular location-specific networks for stomach, liver, and colon cancers. **(A)** Disease network for colon cancer. **(B)** Cytoplasm-specific network for colon cancer. **(C)** Disease network for liver cancer. **(D)** Mitochondrion-specific network for liver cancer. **(E)** Disease network for stomach cancer. **(F)** Nucleus-specific network for stomach cancer.

Disease networks and subcellular location-specific networks for stomach, liver, and colon cancers were constructed by combining protein interaction data, DEGs, cancer genes, and gene subcellular location information (Table 2). The degree distribution for each of these networks approximated a power-law distribution (Figure 3), demonstrating the biological significance of these networks.

**TABLE 2:** Scale of disease and subcellular location-specific networks for stomach, liver, and colon cancers.

Network		Stomach cancer		Liver cancer		Colon cancer	
		Number of nodes	Number of edges	Number of nodes	Number of edges	Number of nodes	Number of edges
Disease		1264	13,806	1378	16,478	1383	6896
Subcellular location-specific	Cytoplasm	812	7592	862	7984	900	3768
	Nucleus	578	6640	663	8440	636	2744
	Endoplasmic reticulum	234	1916	246	2152	234	871
	Mitochondrion	124	388	152	624	132	208
	Golgi apparatus	148	706	148	728	136	375
	Centrosome	69	1096	70	1168	66	378
	Lysosome	47	140	46	176	56	91
	Ribosome	7	28	5	18	16	55
	Peroxisome	11	50	20	154	17	33

### Candidate leader genes from networks

The candidate leader genes of stomach, liver, and colon cancers were recognized as maximum independent dominant sets from the disease networks and subcellular location-specific networks (Table 3).

**TABLE 3:** Number of candidate leader genes for stomach, liver, and colon cancers.

Network		Stomach cancer	Liver cancer	Colon cancer
Disease		529	591	604
Subcellular location-specific	Cytoplasm	340	377	411
	Nucleus	247	298	292
	Endoplasmic reticulum	103	102	104
	Mitochondrion	65	75	64
	Golgi apparatus	73	74	91
	Centrosome	24	24	24
	Lysosome	19	19	22
	Ribosome	3	0	6
	Peroxisome	3	8	8

Expression values of candidate leader genes from each network were used as features for SVM classifiers to distinguish between cancer and normal samples. Classification performance was compared with that of cancer genes (Table 4). Most candidate leader genes from disease networks or subcellular location-specific networks could classify samples better than cancer genes and had a larger size.

**TABLE 4:** Classification performance (AUC) of candidate leader genes.

Genes		Stomach cancer	Liver cancer	Colon cancer
Disease network		0.991	0.996	0.999
Subcellular location-specific network	Cytoplasm	0.991	0.996	0.999
	Nucleus	0.995	0.993	0.999
	Endoplasmic reticulum	0.980	0.996	0.998
	Mitochondrion	0.987	0.994	0.999
	Golgi apparatus	0.985	0.997	0.999
	Centrosome	0.895	0.995	0.999
	Lysosome	0.977	0.983	0.999
	Ribosome	0.846	-	0.969
	Peroxisome	0.644	0.947	0.994
Cancer genes	0.951	0.976	0.993	

### Cancer leader genes from disease networks and subcellular location-specific networks

To reduce the size of candidate leader genes, further classification and enrichment analysis were performed to screen cancer leader genes as candidate leader genes that had good classification performance (AUC >0.75 in the SVM classifier) and enriched in cancer hallmarks (Table 5). Candidate leader genes from some subcellular location-specific networks were not enriched in cancer hallmarks; thus, no leader genes were screened out for them.

**TABLE 5:** Leader genes for stomach, liver, and colon cancers.

Network		Stomach cancer		Liver cancer		Colon cancer	
		Number	AUC	Number	AUC	Number	AUC
Disease		37	0.982	27	0.987	43	0.999
Subcellular location-specific	Cytoplasm	23	0.841	0	-	13	0.994
	Nucleus	31	0.894	0	-	6	0.999
	Endoplasmic reticulum	4	0.930	0	-	10	0.999
	Mitochondrion	7	0.822	9	0.984	0	-
	Golgi apparatus	3	0.862	6	0.985	8	0.999
	Centrosome	9	0.869	4	0.914	0	-
	Peroxisome	0	-	1	0.823	5	0.974

**FIGURE 4:**
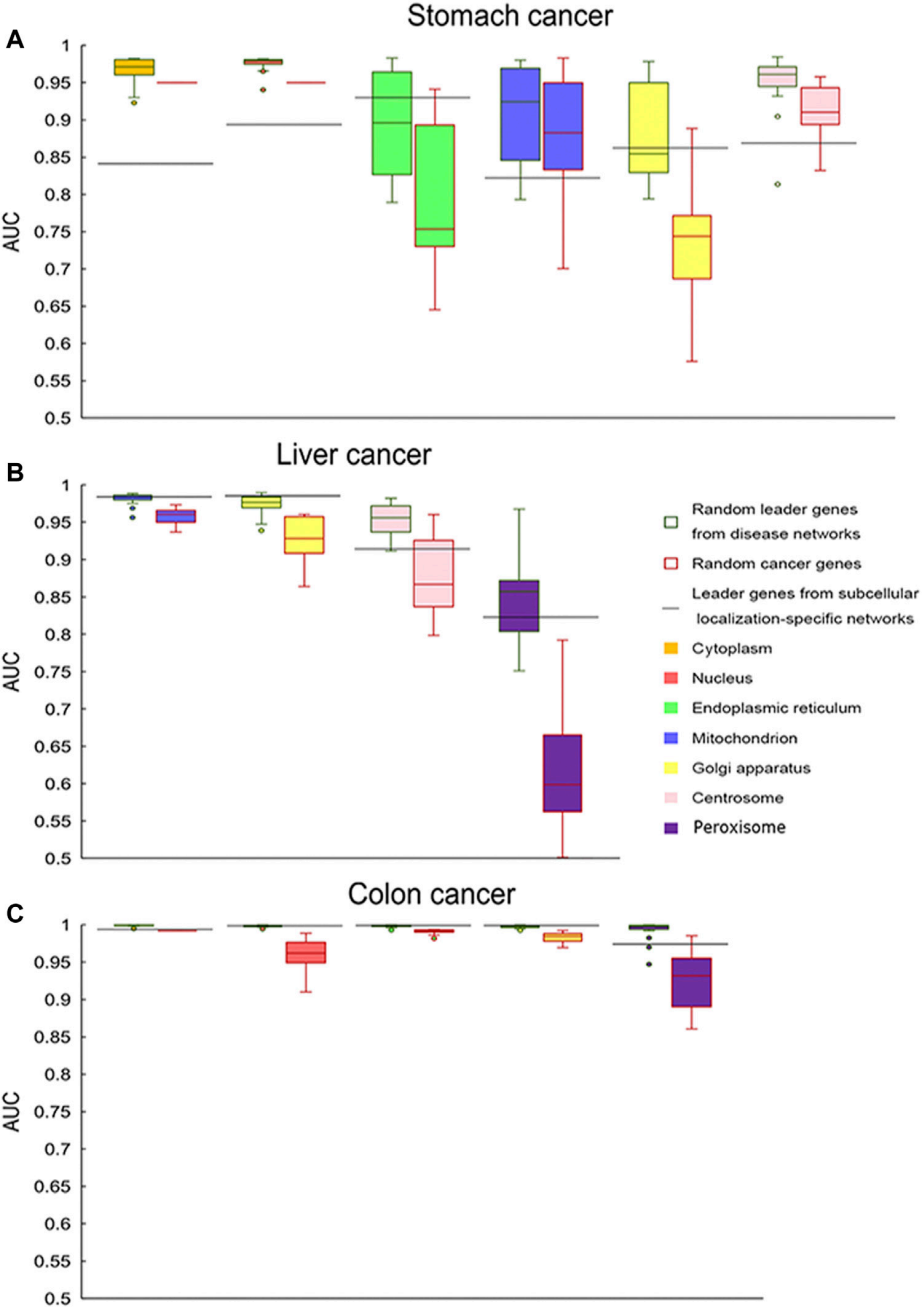
Classification comparison (AUC values) for three types of genes. Leader genes from subcellular location-specific networks are represented by horizontal lines, and random genes from disease networks and random cancer genes are represented by box plots. Different filled colors represent different subcellular locations. **(A)** Stomach cancer, **(B)** liver cancer, and **(C)** colon cancer.

Leader genes from different networks were used to classify samples, and the performance was compared. It was demonstrated that genes from disease networks could classify samples better with a larger size. Cancer genes could also classify samples with better performance and larger size. Thus, random genes with the same number as leader genes from subcellular location-specific networks were selected from disease network leader genes and cancer genes to classify samples. Their classification performance was compared (Figure 4). For liver cancer and colon cancer, the performance of leader genes from subcellular location-specific networks was significantly better than that of cancer genes in all cases and better than that of genes from disease networks in most cases. However, for stomach cancer, only leader genes from the endoplasmic reticulum and Golgi apparatus-specific networks performed better than cancer genes or genes from disease networks.

**FIGURE 5:**
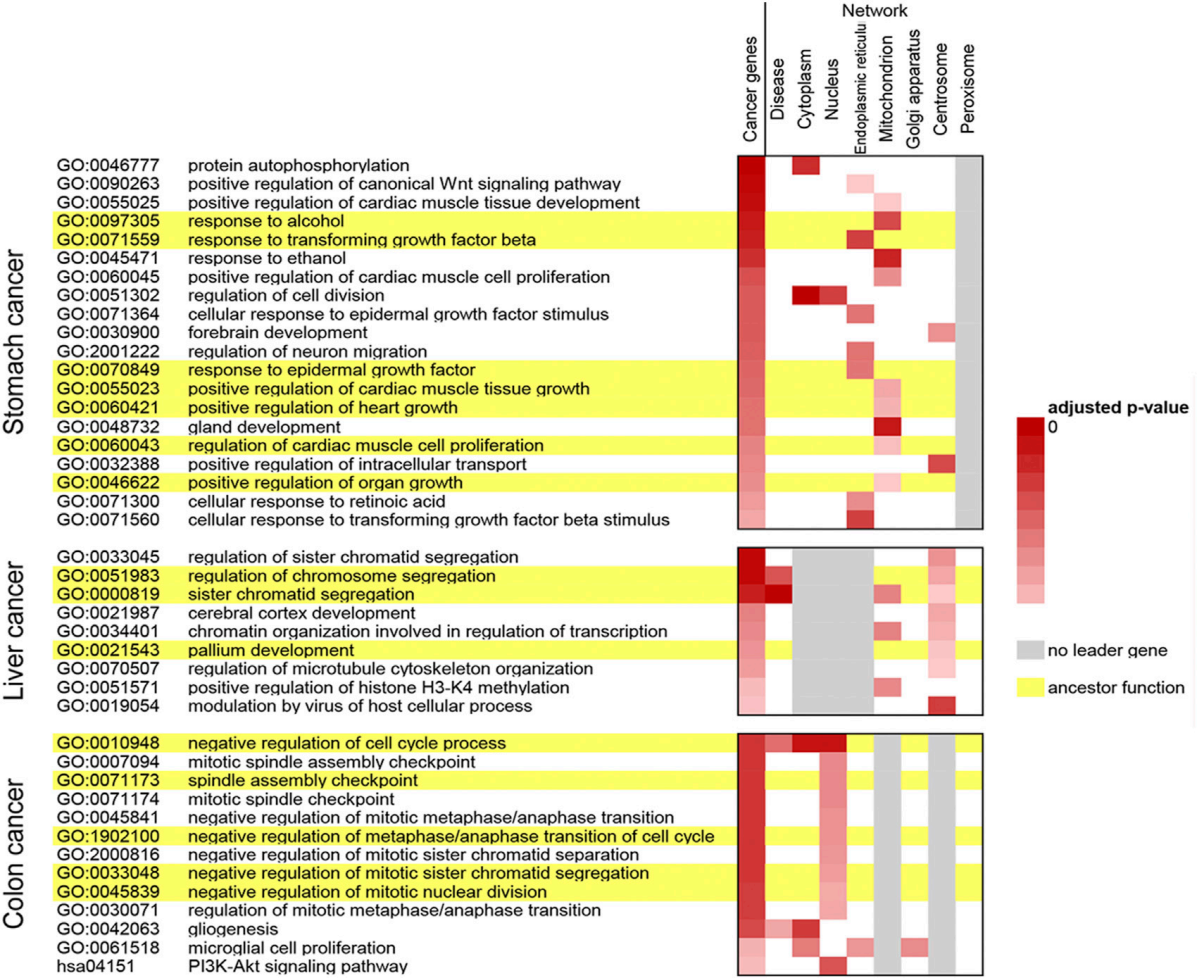
Functions enriched by cancer genes and leader genes from disease networks and from subcellular location-specific networks. Lines in yellow represent ancestor functions, and columns in gray represent networks with no final leader genes.

Functions that were enriched by cancer genes were more related to their corresponding cancer types. Then, functional enrichment analysis was performed for leader genes from disease networks and subcellular location-specific networks (Figure 5). Compared with the leader genes obtained from disease networks, the genes obtained from subcellular location-specific networks were enriched in significantly more cancer-related functions. At the same time, compared with the leader genes obtained from subcellular location-specific networks, the genes obtained from disease networks were mostly enriched in ancestor functions. Therefore, it was speculated that the leader genes obtained from subcellular location-specific networks were more relevant to cancer-related functions.

Furthermore, since each network has its own unique leader genes, the classification performance of unique leader genes from disease networks and from subcellular location-specific networks was compared for each cancer type. It was found that these unique genes either from disease networks or from subcellular location-specific networks had good classification performance (AUC >0.8 in the SVM classifier, Table 6).

**TABLE 6:** Classficiation performance of unique leader genes from disease networks and from subcellular location-specific networks.

Network		Stomach cancer		Liver cancer		Colon cancer	
		Number	AUC	Number	AUC	Number	AUC
Disease		13	0.973	23	0.986	27	0.999
Subcellular location-specific	Cytoplasm	14	0.841	0	-	3	0.984
	Nucleus	15	0.911	0	-	4	0.998
	Endoplasmic reticulum	2	0.849	0	-	7	0.999
	Mitochondrion	7	0.822	7	0.966	0	-
	Golgi apparatus	2	0.818	6	0.985	4	0.989
	Centrosome	4	0.837	2	0.916	0	-
	Peroxisome	0	-	0	-	4	0.970

Functions enriched by the unique leader genes from disease networks and subcellular location-specific networks were also compared. Some obvious differences have been found. For example, the cancer-related functions significantly enriched by unique leader genes from the disease network for stomach cancer, such as DNA metabolic process and DNA replication, were not significantly enriched by the results from subcellular location-specific networks. Meanwhile, functions enriched by unique leader genes from subcellular location-specific networks, including cell cycle process and organelle fission, were not enriched by those from the disease network.

### Final cancer leader genes

**FIGURE 6:**
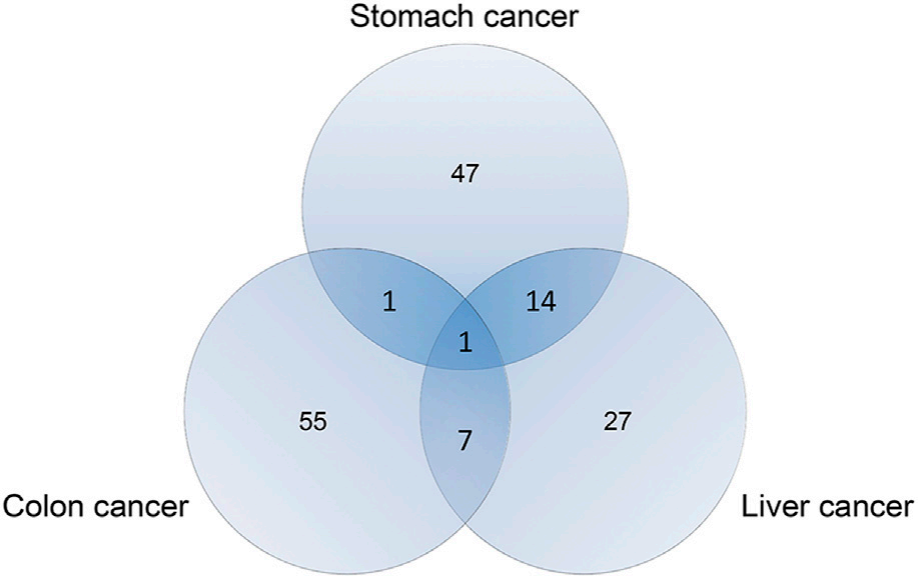
Number of final leader genes for stomach, liver, and colon cancers.

The aforementioned results verified that the leader genes of these networks complement each other. Thus, the final cancer leader genes were all genes identified from subcellular location-specific networks and from disease networks. A total of 69, 43, and 64 leader genes were identified for stomach, liver, and colon cancers, respectively (Figure 6, Supplementary Table S1).

**FIGURE 7:**
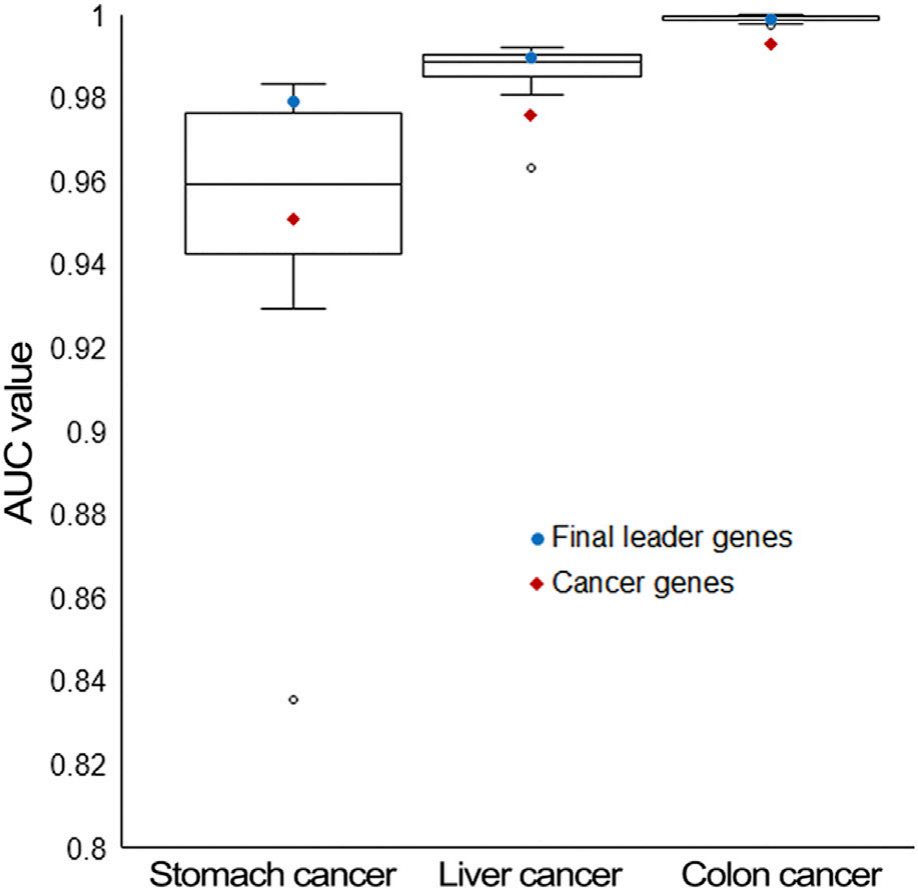
Classification performance comparison for three types of genes. Final leader genes are represented by blue dots, randomly selected final leader genes (the same number as cancer genes) are represented by box plots, and cancer genes are represented by red diamonds.

To show the effectiveness of final leader genes, their performance in classifying cancer and control samples was evaluated, which was significantly better than that of cancer genes. This might be the result of the larger size of genes achieving better classification performance. Therefore, genes with the same number as cancer genes were randomly selected from the final leader genes, and their classification performance was also compared with cancer genes. Final leader genes and random genes had better performance, demonstrating the effectiveness of these final leader genes (Figure 7).

**FIGURE 8:**
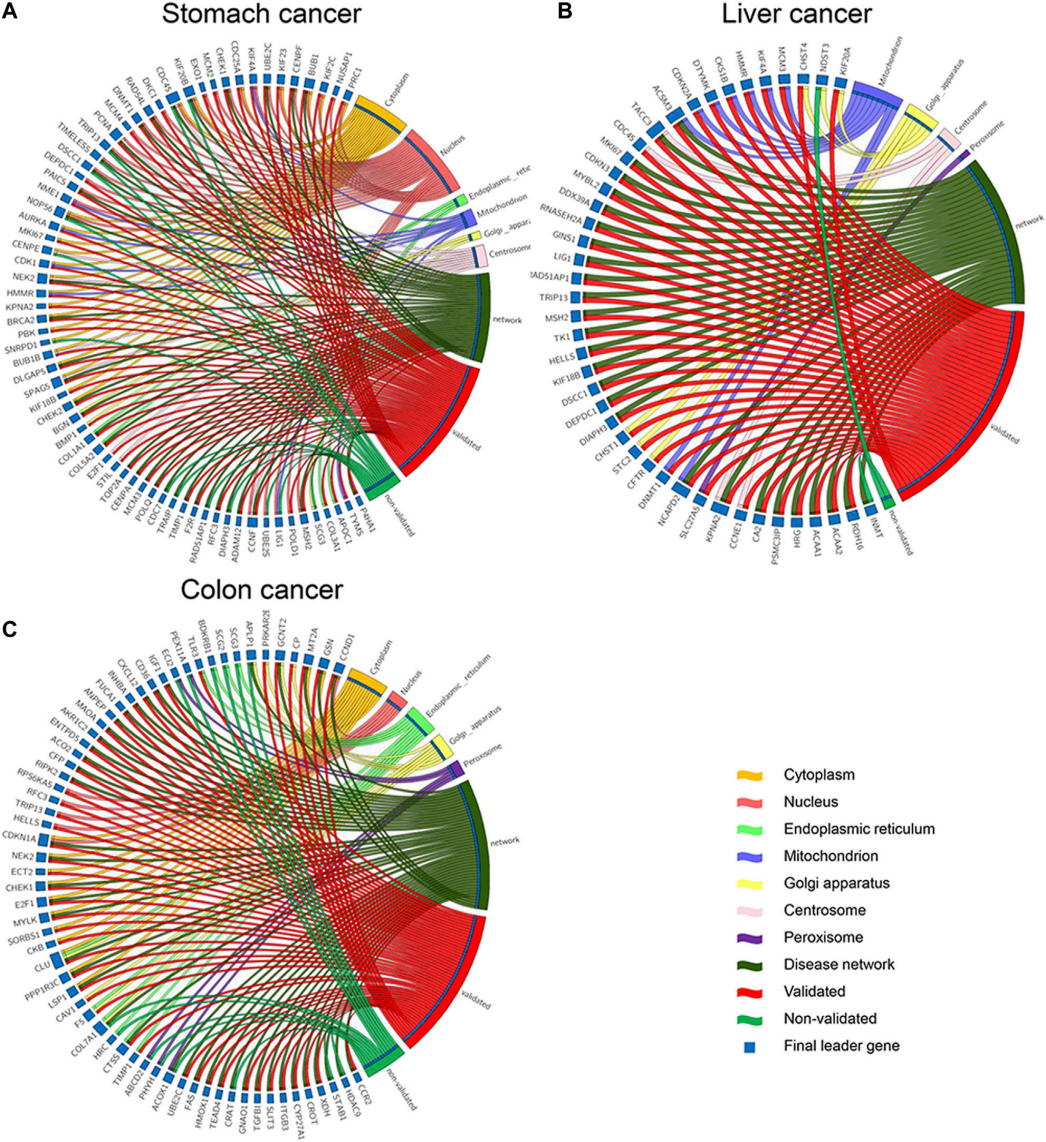
Literature review and subcellular location for final leader genes. Red represents validated and green represents for not validated genes. **(A)** Stomach cancer. **(B)** Liver cancer. **(C)** Colon cancer.

Validation of final leader genes was performed in three aspects, namely, literature review, expression difference for gene products, and classification performance for other datasets. First, a literature review was conducted for the final leader genes of three cancer types, respectively. Of these genes for each cancer type, 92.754% (54/69) were verified to be associated with stomach cancer, 95.349% (41/43) were associated with liver cancer, and 78.125% (50/64) were associated with colon cancer (Figure 8).

For all three cancer types, *TRIP13* mainly located in the nucleus was identified as a common leader gene. Zhu et al. (2019) found that *TRIP13* was the most prominent differentially expressed AAA ATPase gene and a promising candidate oncogene in liver cancer. In addition, the TRIP13 mRNA level was upregulated in peripheral blood in colon cancer tissues, thus making it a potential target for early-stage diagnosis (Soylemez et al., 2021). Nevertheless, no literature showed its association with stomach cancer, and further research is needed.

Twenty-two genes were identified as leader genes across two types of cancers, 16 (72.727%) of which were verified to be associated with corresponding cancers by the literature review. For example, *MSH2* was a leader gene for both stomach and liver cancers. Germline MSH2 X314_splice variants contributed to carcinogenesis, prompting the consideration of other surgery and/or therapy methods for multiple stomach cancer patients (Wang et al., 2020b). Eso et al. (2016) demonstrated that inflammation-mediated dysregulation of MSH2 was a mechanism of genetic alterations during liver cancer development. RFC3 mutation and loss of RFC3 expression may contribute to the pathogenesis of stomach and colon cancers by deregulating DNA repair and replication (Kim et al., 2010), which was concordant with our result that RFC3 was identified as a leader gene for both stomach and colon cancers. Another gene *HELLS* was identified as a leader gene for liver and colon cancers. *HELLS* was reported to regulate chromatin remodeling and epigenetic silencing of multiple tumor suppressor genes in human liver cancer (Law et al., 2019). Alterations in *HELLS* recruitment and function could contribute to the somatic demethylation of SST1 repeat elements undergone before and/or during the pathogenesis of colon cancer (Samuelsson et al., 2017). Previous studies with recent genome-sequencing efforts have revealed there are a limited number of large-effect genes that participate in multiple cancer types (Rickel et al., 2017), which was consistent with our results. These common leader genes indicated that these three types of digestive system cancer might be associated with or have some common pathogenesis.

Other genes act as specific leader genes in one cancer type, and 82.171% of them were found related to a corresponding cancer type in the literature. Shimura et al. (2020) suggested that urinary levels of *ADAM12* were a significantly independent diagnostic biomarker for stomach cancer, and a urinary biomarker panel containing *ADAM12* significantly distinguished between cancer patients and normal samples. *TACC3* promotes stemness and is a potential therapeutic target in hepatocellular carcinoma (Zhou et al., 2015). *GNAO1* was found to be differentially expressed in colon cancer compared to normal samples and might be a potential colon cancer biomarker (Hauptman et al., 2019). These specific leader genes indicated that although these three types of digestive system cancer might be associated with or have some common pathogenesis, their pathogenic mechanisms still have many differences.

Some final leader genes were identified from multiple subcellular location-specific networks. One final leader gene for stomach cancer, *CDC45*, was identified from three subcellular location-specific networks and the disease network. Its main subcellular locations were the cytoplasm, nucleus, and centrosome. Two previous studies have found that *CDC45* could promote some cancers by co-expressing with other genes (Hu et al., 2019; Lu et al., 2021). One final leader gene for colon cancer, *CLU*, was identified from the cytoplasm, endoplasmic reticulum, Golgi apparatus-specific networks, and the disease network. A previous study has found that the high mRNA expression level of CLU in colon cancer patients indicated a poor prognostic outcome (Artemaki et al., 2020).

**FIGURE 9:**
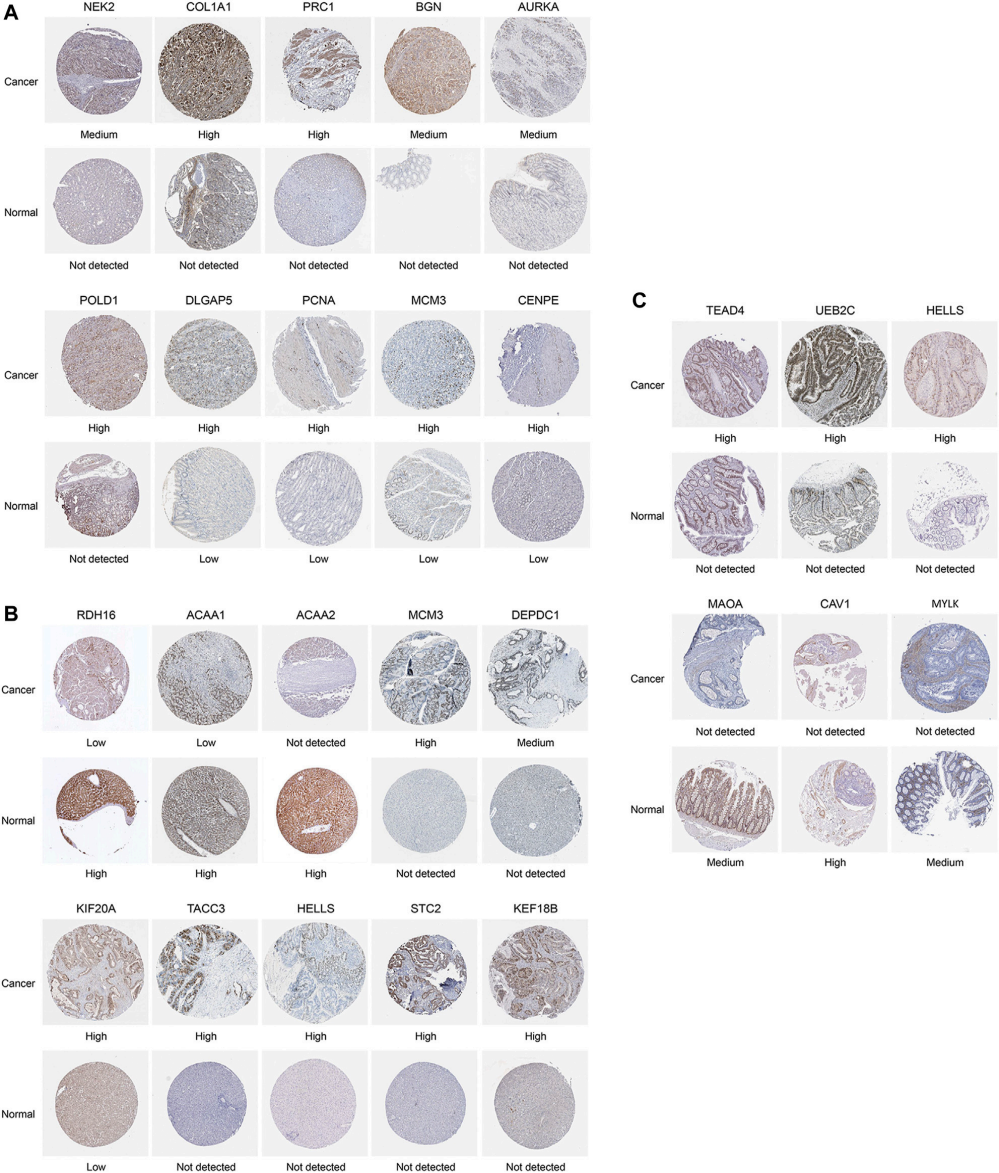
Images for part of final leader gene immunohistostaining between cancer tissues and normal tissues in the Human Protein Atlas database. **(A)** Stomach cancer. **(B)** Liver cancer. **(C)** Colon cancer. High, medium, low, or not detected represent the expressional levels of the gene products, whose difference meant expression difference for gene products.

Then, to demonstrate the clinical significance of final leader genes, we performed the analysis with experimental data from HPA and GEO. Alteration of protein expression levels for products of these final leader genes in cancer and normal condition was assessed. Expression levels for proteins encoded by final leader genes were obtained from the HPA database, in which 11, 5, and 9 final leader genes were not found for stomach, liver, and colon cancers, respectively. For other final leader genes, 39, 34, and 26 final leader genes for stomach, liver, and colon cancers, respectively, obtained from the HPA database showed the same up/downregulation direction as those from TCGA data (some significantly up/downregulated ones are shown in Figure 9), such as *COL1A1*, *MCM3*, and *CAV1*. Proteins encoded by *HELLS* were not expressed in normal tissues, whereas high expression levels were observed in both liver and colon cancer tissues. Protein expression levels of 13, 3, and 8 final leader genes for stomach, liver, and colon cancers, respectively, also changed between cancer and normal statuses, while they had an opposite regulation direction to TCGA.

Furthermore, expression values of final leader genes of three cancer types were used as features for SVM classifiers to classify samples from independent GEO datasets of different platforms for further validation. All final leader genes could distinguish between cancer and normal samples with high performance (AUC >0.95) (Table 7).

**TABLE 7:** Classficiation performance of final leader genes for GEO datasets.

	GEO dataset	Platform	AUC value
Stomach cancer	GSE118916	GPL15207	0.955
Colon cancer	GSE44076	GPL13667	0.999
Liver cancer	GSE76427	GPL10558	0.952

Validation of the aforementioned three aspects exhibited that part of final leader genes for stomach, liver, and colon cancers were related to corresponding cancer types. Since all of these genes could be used to classify different samples, genes without literature or altered regulation direction were also disease-related and could be used as diagnostic biomarkers. Further studies are still needed.

Moreover, the final leader genes identified by our integrated procedure were compared with the result genes of methods from Bailey et al. (2018), Ding et al. (2021), and Liu et al. (2022) according to the AUC values of SVM classifiers. The results of our genes and other methods all had good classification performance with no significant difference in terms of classification performance (Table 8). However, most genes of final leader genes could not be obtained by other methods, which were performed from different aspects, such as *CDC45* and *CLU*. Therefore, the comparison with several latest methods demonstrated that the final leader genes identified by our integrated procedure would contribute to future digestive cancer research studies.

**TABLE 8:** Classification performance of the result genes used as features of SVM of the algorithm in this article and the latest existing algorithms.

	LIHC	COAD	STAD
Bailey et al	0.994010695	0.998831301	0.940333333
Liu et al	0.995775401	0.931046748	0.917083333
Ding et al	0.88	-	0.730
Final leader genes	0.98973262	0.99898374	0.97925

## Discussion

Colon, liver, and stomach cancers are among the top five most common causes of cancer death in the world. In this article, leader genes for these three digestive system cancer types were identified by our proposed procedure. First, disease and subcellular location-specific networks for these three cancer types were constructed, respectively. Candidate leader genes were recognized in these networks. Then, candidate leader genes enriched in cancer hallmarks and disease-related functions and able to distinguish between cancer and normal samples were identified as final leader genes.

In addition to classification performance for cancer and normal samples, the value of cancer genes manifests in predicting overall survival to reflect tumor progression at the molecular level, achieve individualized survival predictions, and guide clinical management. Thus, a univariate Cox regression analysis was performed to investigate the association between each final leader gene and the prognosis for patients with survival and clinical information in TCGA. About half (Hauptman et al., 2019) of stomach cancer final leader genes, most (65) of the liver cancer final leader genes, and only two colon cancer final leader genes were found to be significantly associated with survival (*p* < 0.05). The reason for some final leader genes identified in this article not being appropriate for prognosis prediction is probably that the networks were constructed using DEGs between cancer and normal samples rather than other prognosis-related genes. Taking prognosis into consideration and identifying prognosis-related genes will be a concern in our research in the future.

Unique genes from disease or subcellular location-specific networks have a good ability to distinguish between cancer and normal samples (AUC>0.8). However, these unique leader genes could not be obtained from other networks, which indicated that the leader genes obtained from different networks have a certain complementarity. These unique leader genes are involved in the occurrence of cancer and have their own unique role according to the networks they are located in. This could provide a certain reference for understanding the underlying mechanism of each subcellular region in cancer occurrence at the cellular level. The final leader genes obtained in this article are the union of the leader genes obtained from disease and subcellular location-specific networks. These genes could distinguish between cancer and normal samples with good performance (AUC>0.8). Furthermore, literature reviews, protein expression levels, and independent datasets all verified their association with corresponding cancer types. These final leader genes of digestive cancers are vital for understanding the molecular mechanisms responsible for the onset of these disorders. Identical genes may indicate the same pathogenesis.

A total of 69, 43, and 64 final leader genes for stomach, liver, and colon cancer, respectively, were identified in this article. Based on the Disease Ontology (https://disease-ontology.org/) database, six genes in the final leader genes of colon cancer were confirmed to be involved in the occurrence of colon cancer, such as *CCND1*, whose overexpression significantly increased the progression of cancer (Li et al., 2021), and *CDKN1A*, which is a tumor suppressor of colon cancer (Halaburková et al., 2017). Similarly, there are 8 genes in the final result of lung cancer that were confirmed to be involved in important processes in cancer. For example, *DNMT1* interacted with *NEAT1* to regulate cytotoxic T-cell infiltration in lung cancer *via* inhibition of the cGAS/STING pathway (Ma et al., 2020). Also, abnormal expression of *CCNE1* is associated with cell cycle dysfunction (Zheng et al., 2019). Five genes in the final leader genes of stomach cancer play important roles in cancer. For instance, *MKI67* is a potential indicator to predict the prognosis of patients with stomach cancer and identify high-risk cases (Guo et al., 2018), and *TYMS* may be potential biomarkers for prognosis and chemotherapy guidance for stomach cancer (Cao et al., 2017).

Although subcellular location information from databases was used to construct subcellular location-specific networks in this article, the subcellular location of genes/proteins might change or be influenced by many factors, affecting their functions and leading to diseases, including cancers. For example, colon cancer has been found related to many subcellular translocations of proteins (Hauptman et al., 2019). Therefore, early diagnosis of cancers can rely on not only the known subcellular location information of genes/proteins but also the change of subcellular locations between normal and cancer cells. Further research studies on subcellular location change will be essential to reveal other cancer leader genes.

Moreover, this is a study based on existing datasets using bioinformatics. A graph theory-based algorithm was used to recognize candidate leader genes in this study. In addition to the leader genes in this article, some genes might not only directly participate in cancer-related functions but only indirectly affect the expression of other cancer-related genes through the protein interaction network. Further extraction and study of these genes are also important for complementing the leader genes and understanding the underlying mechanisms of cancer in protein interaction networks. Our understanding of the roles these final leader genes play in the genesis of these cancer types is in its infancy. Some results require further experimental verification. We hope that there will be other databases and a large number of experiments to verify the feasibility of these final leader genes in the future and provide a reliable predictor and therapeutic target for digestive system cancer patients.

## Conclusion

In summary, our study identified leader genes for three digestive system cancers, stomach, liver, and colon cancers, by application of a proposed graph theory-based algorithm to human PPI networks based on subcellular location information and further considering cancer-related characteristics. These final leader genes were believed to be an early signal in human carcinogenesis and to be potential cancer biomarkers. The integrated procedure proposed in this article for identifying cancer leader genes could be expanded to shed light on the mechanisms, early diagnosis, and treatment of other cancer types.

## Data Availability

The original contributions presented in the study are included in the article/Supplementary Material, and further inquiries can be directed to the corresponding author.
